# Implementation of clinical research trials using web-based and mobile devices: challenges and solutions

**DOI:** 10.1186/s12874-017-0324-6

**Published:** 2017-03-17

**Authors:** Roy Eagleson, Luis Altamirano-Diaz, Alex McInnis, Eva Welisch, Stefanie De Jesus, Harry Prapavessis, Meghan Rombeek, Jamie A. Seabrook, Teresa Park, Kambiz Norozi

**Affiliations:** 1 Faculty of Engineering, Western University, London, Canada; 2 Department of Paediatrics, Western University, London, Canada; 3 Children’s Health Research Institute, London, Canada; 4 Paediatric Cardiopulmonary research laboratory, London Health science centre, London, Canada; 5 School of Kinesiology, Western University, London, Canada; 6 Brescia University College, Western University, London, ON Canada; 7 Department of Paediatric Cardiology and Intensive Care Medicine, Medical School Hannover, Hannover, Germany; 8 Department of Paediatric Cardiology and Intensive Care Medicine, University of Goettingen, Goettingen, Germany; 9 Department of Paediatrics, Division of Paediatric Cardiology, Western University, 800 Commissioners Rd E, PO Box 5010, London, ON N6A 5W9 Canada

**Keywords:** Web-based technology, e-health, Privacy, Security, Obesity, Paediatric cardiology

## Abstract

**Background:**

With the increasing implementation of web-based, mobile health interventions in clinical trials, it is crucial for researchers to address the security and privacy concerns of patient information according to high ethical standards. The full process of meeting these standards is often made more complicated due to the use of internet-based technology and smartphones for treatment, telecommunication, and data collection; however, this process is not well-documented in the literature.

**Results:**

The Smart Heart Trial is a single-arm feasibility study that is currently assessing the effects of a web-based, mobile lifestyle intervention for overweight and obese children and youth with congenital heart disease in Southwestern Ontario. Participants receive telephone counseling regarding nutrition and fitness; and complete goal-setting activities on a web-based application. This paper provides a detailed overview of the challenges the study faced in meeting the high standards of our Research Ethics Board, specifically regarding patient privacy.

**Conclusion:**

We outline our solutions, successes, limitations, and lessons learned to inform future similar studies; and model much needed transparency in ensuring high quality security and protection of patient privacy when using web-based and mobile devices for telecommunication and data collection in clinical research.

**Electronic supplementary material:**

The online version of this article (doi:10.1186/s12874-017-0324-6) contains supplementary material, which is available to authorized users.

## Background

The use of web-based applications and smartphones is becoming more prevalent in healthcare and clinical research settings. The various uses of internet and mobile technology include: health information systems in hospitals; outpatient monitoring, such as wearable telemetry systems (Body Scan Networks) that measure physiological changes in patients with chronic illnesses [1, 2]; telecommunication between patients and healthcare professionals, such as tele-counselling and text reminders for mental health patients [3–5]; and data management systems for clinical research studies [6]. There are many benefits of web-based and mobile technologies, including continuous monitoring for chronically ill patients, better quality care and feedback, reduced hospitalization time, increased medical capacity, and reduced medical cost [1, 2, 7]. However, amidst the rapid spread and progression of technology in healthcare, we must uphold high ethical standards for protecting patient privacy. Kotz et al. cautions that designers and developers of healthcare information technologies must address security challenges; otherwise, the benefits for healthcare information technology (IT) will be elusive [8]. The very nature of the internet introduces security and privacy issues, including potential privacy breaches through hacking and data corruption during transfer [9]. A review by Seko et al. revealed that ensuring confidentiality and privacy was the most commonly stated concern in published studies regarding mobile mental health interventions for adolescents [3].

Despite the ever-present security concerns, the process of addressing these concerns, especially in the development and implementation of web-based, mobile health interventions, is not well-documented and fragmented in literature. Literature is fragmented in terms of techniques and schemes that are used, such as data encryption, anonymization, and pseudonymization techniques. Also, few studies report the entire process of gaining ethics approval regarding patient privacy or how different frameworks and techniques are used specifically in clinical research to develop and implement a web-based mobile intervention. Clinical research studies implementing web-based mobile health interventions do not often discuss data security in detail. Those that do report security measures for collected data most commonly report having secure servers/institution firewalls, username/password authentication, 128-bit data encryption, and de-identification of personal information. Each intervention is unique, with different methods for interacting with participants and requiring tailored safeguards for protecting patient privacy. Without a standardized procedure for implementing web-based and mobile systems in clinical research, it is important to demonstrate transparency regarding security and protection of patient privacy when conducting such studies. This is not only to inform other researchers, but to demonstrate ethical duty to privacy.

## The “Smart Heart” Trial

The Smart Heart Trial is an ongoing single arm feasibility study to examine how a 12-month lifestyle intervention will impact health and well-being measures, such as body weight and body composition, in overweight and obese children and adolescents with congenital heart disease (CHD) in Southwestern Ontario. All participants are provided with a complimentary smartphone and 1-year mobile plan. The intervention consists of nutrition and fitness counseling provided over the phone by health coaches (dieticians and fitness specialists). There is also a web-based component, which provides participants with optional e-mails for communicating with their health coaches and a goal-setting application introduced 6 months into the program for recording daily activity and nutrition behaviours. Health coaches have administrative access to the web application in order to follow and comment on each participant’s progress. The data collected is used to inform health coaches in their practice and to evaluate the web-application (patterns of use) and define participant engagement in the program. There was an unexpected delay in gaining ethics approval for this study, for there were concerns regarding the web-based component and potential risks for patient privacy. In addressing the privacy issues raised by our research ethics board, we strengthened our security measures and recognized the importance of protecting patient privacy when implementing web-based applications for clinical research.

## Ethical standards and legislation regarding patient privacy in Canada

In Ontario, Canada, all healthcare practices must adhere to the Personal Health Information Protection Act (PHIPA), a provincial law based on 10 privacy principles: 1) Accountability for personal health information (PHI); 2) Identifying purposes for the collection of PHI; 3) Consent for the collection, use, and disclosure of personal information (PI); 4) Limiting collection of PHI; 5) Limiting use, disclosure, and retention of personal information (PI); 6) Ensuring accuracy for PHI; 7) Ensuring safeguards for PI; 8) Openness about PI policies and practices; 9) Individual access to own PI; and 10) Challenging compliance with the hospital’s privacy policies and practices [10]. All research involving human subjects in Canada must adhere to the Tri-Council Policy Statement: Ethical Conduct for Research Involving Humans (TCPS2), a joint, federal policy of the Canadian Institutes of Health Research (CIHR), the Natural Sciences and Engineering Research Council of Canada (NSERC), and the Social Sciences and Humanities Research Council of Canada (SSHRC) [11]. The policy’s section on Privacy and Confidentiality states that researchers have an ethical duty of confidentiality, safeguarding information entrusted to them without misusing or wrongfully disclosing it [11]. The Smart Heart Trial underwent ethics review by two organizations, London Health Sciences Centre (LHSC) and Western University, ensuring the study met PHIPA, TCPS2, as well as hospital policies and ethical standards.

## Objectives

The objective of this paper is to provide a detailed overview of the challenges the Smart Heart Trial faced in meeting the high standards of the research ethics board (REB), specifically regarding patient privacy. We outline our solutions, and describe their successes and limitations, in order to inform future similar studies and model much needed transparency in ensuring high quality security and protection of patient privacy when using web-based and mobile devices for telecommunication and data collection in clinical research.

## Addressing privacy issues for smart heart trial

### Disclosure of information regarding Web application security measures

The first REB request was a full description of the web application and how it protected patient privacy. Thus, we provided information on: 1) data type and delivery, including study population, data storage locations, logging, and data retention; 2) privacy, including information security, access to information, disclosure, and consent; and 3) security, including hosting environment and authentication. This information is summarized in Table 1.

**Table 1:** Description of Web-Based Application for the Research Ethics Board (REB)

*Section 1 - Data Type and Delivery*	
Study population	Obese patients (ages 7-17) with existing cardiac condition
Data Storage Locations	1. LHSC Shared Drive: Identifiable data is stored only on the LHSC network on a private shared storage location. • Type of Data: • Notes and documentation gathered from the health coaches, demographic data, etc. • Patient demographic data. • Study related documentation. • Database backups and Web access logs. 2. Web Server: Anonymized data is maintained on ISQ Solutions Inc. web server. This data is stored in SQL DB with user authentication via a web interface or mobile phone app. All information is entered under a generic patient login name (e.g., ‘patient1’, ‘patient2’, etc) and password. • Type of Data: • Numerical data related to the users’ exercise and eating habits. 3. Email System: Data is also transferred via email to and from patients, again using anonymized generated patient ID. Information is transient and is deleted as the trial data is analyzed. • Type of Data: • Follow-up from health coaches will be transferred to and from patients. 4. External Drive: The identified data stored on the LHSC network is backed up with the standard LHSC backup utility, and copied to an external encrypted HD that is stored on site at LHSC in a locked cabinet, compatible with hospital records-keeping standards. • Type of Data: • Notes and documentation gathered from the health coaches. • Patient demographic data. • Study related documentation.
Logging	• The phone and email communication will be logged on pen-and-paper forms by the health coaches as it is acquired. • Access to the web site and database will be logged to an activity log and stored on the web server. This log is backed up nightly to the LHSC server shared area.
Data Retention	• All study data will be maintained five years after the study has been completed, as per hospital protocol.
*Section 2: Privacy*	
• Only information stored on the LHSC network contains patient identifiable information. All other information is entered under a generic ID with password.	
Information Security	• External access to data: • Anonymized information is stored on an external web server. Web server requires ID and password for access. • Email communication to and from health coach. Emails are stored on external email server. Anonymized accounts are used, and no patient identifiable information is transferred. ID and password are required for access. • External access to the shared drive on LHSC server is accessible to team members only via Juniper VPN, utilizing 2 form authentication. • External data backup drive: • Kept onsite at hospital, stored in a locked drawer, and encrypted with 512-bit encryption and 64 character password. Trucrypt is used to encrypt the drive. • System Tracking, backup and logging: • Web site access is tracked and logged. • Email access is logged. • Website, database, all log files are backed up nightly. • Shared Drive is backed up nightly and archived to encrypted external drive.
Access to Information	• Role-based access • Access rights (e.g., read only, read/modify) to web site, shared drive, and e-mail account is controlled depending on type of user (physician, health coach, participant, vendor/ISQ Solutions. Inc, technical support staff)
Disclosure	• No personal health information will be disclosed to any persons who are not employees or agents of the hospital.
Consent	• Patient/SDM consent is being obtained for the collection, use and/or disclosure of the information for the study.
*Section 3: Security*	
Hosting and Environment	• LHSC Shared Drive is stored on a server in the LHSC Data Centre. • Web Server is hosted by ISQ Solution Inc. o Windows2008 server o SQL2008 Database. o IIS 7 o Backend access is via sftp or https. • Email Server is hosted by ISQ Solution Inc. o Windows2008 server o Web Mail • Secure web mail client available. (https)
Authentication	• All access requires an ID and password. • No information is stored on the phone or workstation. • All data accessible via email or web server and has no patient identifiers. • System access is logged.

Despite these safeguards (Table 1), the REB required further information which caused a delay in the initiation of the study, due to a number of special issues associated with the use of web-based services to gather the anonymized data. We recognized the severity of the following issues raised by the committee, and accordingly, we prepared the following response to the specific items raised by the REB. As such, this information may prove valuable in assisting other researchers with navigating policies of their own REB.

Ontario’s “PHIPA” privacy laws are based on 10 privacy principles. Of these principles 1 to 4 pertain to the “*Accountability, Collection, Consent, and Constraints and Limits”* on data collection. These are addressed through non-technical means, through the protocols of the study and the interactions with participants, as governed by the Research and Ethics Protocol of our study; these are general administrative principles. Similarly, principles 5, 6, 8 and 10 regard policies that limit the use and restrict disclosure of records, as well as ensuring accuracy and openness of these policies. Principle 9 deals with “individual access” to records. Since our patients enter their own data, and no other information is stored, they have first-hand knowledge of this information. No mechanism is provided to enable participants to directly access the recorded information once it is entered. However, participants are able to access to their own data in order to review their progress, as part of the experimental protocol, by contacting the Study Consultant. What remains to be addressed is principle 7, which deals with *“Ensuring Safeguards*” for participants PI, and is the focus of the remainder of our report.

### Addressing the general vulnerability of Web-based services

We were asked by the REB to address a number of generic concerns regarding the general security of web services. This is not an unreasonable request; yet many of the REB’s concerns were couched in technical terms such as ‘minimization of attack surface’ and other web security idioms. The techniques we utilized for re-deploying our web application prototype intrinsically allowed for a rich layer of security. To be sure, most IT teams, even the ones who actively engage in threat modeling, do not understand their web application’s attack surface. From an architectural standpoint, it is typical for such teams to brainstorm with a whiteboard, and create a high-level diagram of all the major components and how they interact. From the source code perspective, you can examine the dependencies between files and what database permissions are needed. One can even point to the encryption scheme used by our internet services provider. For complex systems, this exercise can provide a complete picture of the processes, data flows, protocols, privilege boundaries, external entities, and so on, which would provide you with an understanding all of the potential attack vectors. However, in our particular case, the interactions were not complex and quite straightforward, without interactions between service-oriented modules.

To address these security concerns we employed a defence strategy using coordinated protective layers in combination with arranging the defence components in ways that are complementary and co-supportive. This is exactly the sense in which it is used for security and safety precautions -- to concede that no single defence can be perfectly reliable. Typically, this involves using multiple passwords, anti-virus software, secure server technology and internet firewalls. We employ these and make use of *Logging and Sandboxing* -- the recording of all interactions, and ensuring that no general “shell script” functions are enabled along with the data records, respectively. From that top-level perspective, we describe our approach to defence and security.

#### Minimization of the attack surface

**Fig. 1:**
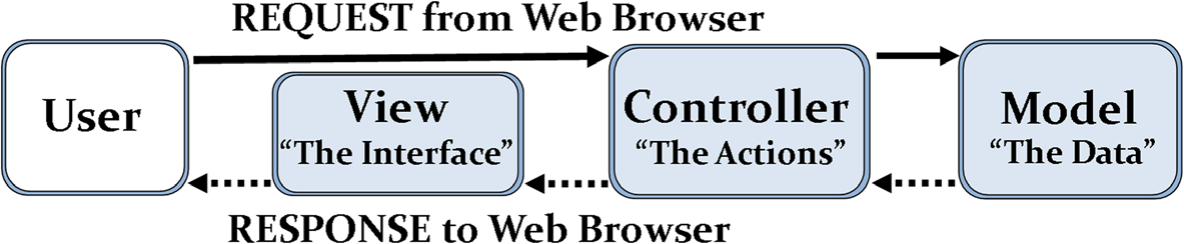
Separation of Model, View, and Controller (MVC) for web-based systems. MVC is a pattern for developing applications that contains: Models which represent the data of the application, Views which are visualize representations of the data, such as dynamically generated HTML responses, and Controllers that connect the two by functioning to handle incoming browser requests, retrieve model data and return responses to the browser

It used to be that web application security considerations were restricted to concerns over the number of ports that would be open on a server. Modern operating systems have fully specified firewall rules implemented. In the creation of our SQL database, we do not allow other web services to access this database. We make use of the secure *Entity Framework* to display data from the single table on a page when requested by the authorized administrator of the project; and this is handled by the Microsoft secure framework and the *Model, View, Controller* (MVC) software pattern (Fig. 1).

#### Use of defense in depth

**Fig. 2:**
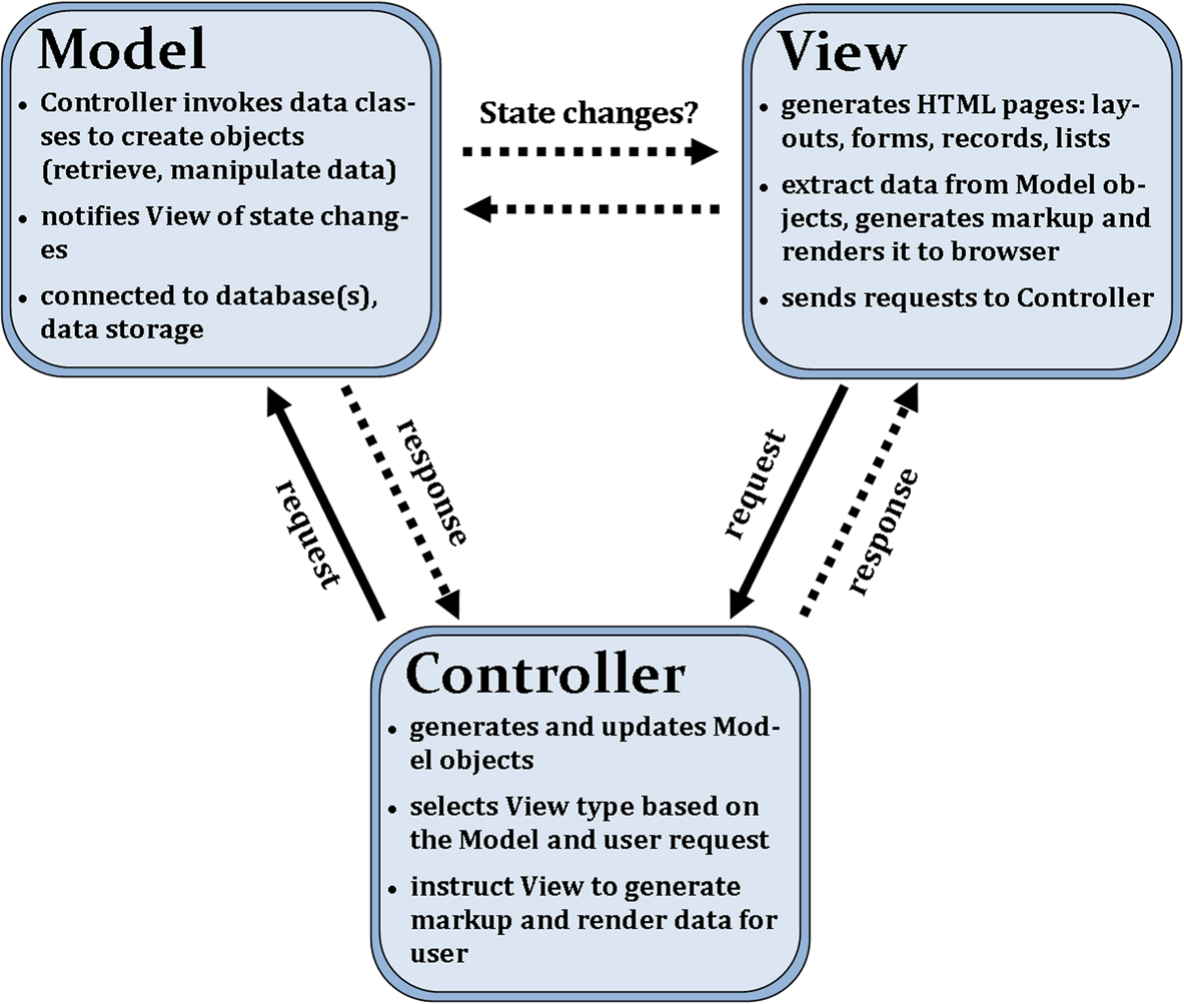
Breakdown of functions and message passing in classical MVC. This figure describes some of the basic functions of each of the three components of the classical MVC pattern. Separating these roles into three separate components makes the system easier to develop, test, maintain and update. All of which enhances the security of the application and system

Web Service developers typically make use of pre-existing service libraries; following the installation instructions to define and map two servlets into the web.xml file, and then to integrate with the web app. After a bit of educated trial and error, it may become functional. This is where most developers stop. Unseasoned developers might need to make use of a web “action” parameter which can be function-typed as either “view”, “edit”, or “delete”; and what if their application only uses “view”? They would still be exposing the other actions for probing by anybody who knows the URL syntax for that API. In our development, we have considered the critical question: “How much functionality do we actually need?”. Our application makes straightforward use of HTTP *get* and *post* with validation methods. We have provided full implementations of the API methods for forms posting and database queries, thus eliminating these attack modes. We make use of input validation when the forms are posted, and we have full access to our website files at all times, addressing the final question on fixing code directly (Fig. 2).

“*Defence in Depth*” is the old military strategy of arranging protective layers in a coordinated fashion -- the goal is to slow the advance of the enemy, rather than to suppose that one fail-safe line can ever be established as an absolute wall to the attack. *S*ecure systems need to be developed according to the principle that each application layer and sub-system is responsible for its own security. Each level should function as its own gatekeeper and act as if it is always interacting directly with the outside world, authenticating and authorizing users before allowing them to perform any actions. Our design employs this methodology through the use of web authentication, which utilizes anti-forgery tokens for each transaction between client and server.

#### Use of least privileges

Our Web Application handles input requests from the users by executing them with the least amount of privilege. We have designed our application not to require elevated rights and avoid scenarios that require them. When required, they would be temporary and restricted by granting them for only the minimum time required to complete the task followed by immediate removal. There is also no disk access, and consequently, files cannot be deleted, uploaded or executed.

#### Employment of secure defaults

Starting with version 1.1, ASP.NET has built-in input filtering for implementation of secure defaults. Any attempt to submit a request containing bracketed tags (“<“or”>”) in any of its form data, query string parameters, or cookies, results in an error page indicating that malicious input has been detected. This, in addition to our server-side validation, prevents malicious attack through the HTTP request. We also make use of *SQL Membership*, which is a “secure default” implementation for the *Database Access* side.

#### Assumption that the external systems are insecure

On a web-based system, any input from a user’s browser, or another system, should always be treated as a potential threat. Our design imposes validation for each and every interaction processed by the Web Application. We never assume that we can trust the HTML request simply because it has already been validated elsewhere. For example, when a user types an entry into a web form, the client-side code (e.g. Javascript) can validate the data to ensure it complies with the range of acceptable values. This may help to create a more robust user experience, but this is certainly not our only line of defence. It is very easy for a would-be attacker to submit a form post directly to the server, thus bypassing any client-side validation. Therefore, our design employs server-side verification. We make use of controller-based procedures to validate the data, thus blocking the use of web-based attacks that circumvent the client-side validation. For web maintenance and development, our server provides a secure connection between a users’ computer and their services that protects e-mail, data, and uploads. In order to establish connections, they make use of a secure socket layer (SSL) (see Additional file 1).

## Successes, limitations, and lessons learned

The Smart Heart Trial was approved in June 2012 by delegated review of the Clinical Research Impact Committee of the LHSC and Western University (REB #18843).

Avancha et al. identified misuse of patient identities, unauthorized access to PI, and unauthorized disclosure of PI as potential threats to user privacy; and suggests authentication, anonymity, consent, and access control as security measures against these threats [7]. The Smart Heart Trial’s web-based application employs these protective measures. For example, in one hypothetical scenario an outsider gains access to de-identified data and can then re-identify patients from anonymized research data. In the Smart Heart Trial, de-identified data is only stored on the hospital network in a private shared storage site. Access to the shared drive is secured by controlling user access rights and individual passwords, which must meet up to 3 requirements (e.g., one upper case letter) for added password complexity. There is also a virtual private network (VPN) available to the project researchers for data access, but user rights access and password controls follow the same stringent rule set. Thus, the chances of an outsider obtaining de-identified data are very slim. A breach of privacy would require auditing of group membership. Anonymized data is stored on a secure, external web server hosted by ISQ Solutions, Inc., and only contains numerical data that would be meaningless without the knowledge of the health coaches and physicians associated with the study. Patients are assigned generated usernames (e.g., patient 1, patient 2) and passwords for logging in to the web application. They cannot view previously submitted data. Thus, even if an outsider obtained the correct patient login information, they cannot access anonymized or de-identified information, as no information is stored on the phone or workstation. Informed consent regarding privacy policy and the collection, use and/or disclosure of information for the study was obtained from patients or parents/guardians of minor participants. As part of the recruitment process, and prior to consent, participants or their parents/guardians were verbally informed of the inherent risks associated with electronic PHI data storage and our obligations to maintain their privacy. The study also employed role-based access restricting access to only legitimate personnel and minimizing the possibility for intentional or accidental modification of PI [7]. For example, in the Smart Heart Trial, participants are granted “read only” rights to the web application, and no rights to access the hospital shared drive.

A limitation of the Smart Heart Trial’s web application is that a user has unlimited tries to log in to the web application. However, the user is not informed whether their error is in the username or password, rendering automatic username/password generators ineffective. Furthermore, there were “trade-offs” between web application functionality and security—the more complex the application, the greater the security risks. Initially, we wanted to implement a complex feedback system between participants and health coaches where participants can view their progress to date. However, allowing patients to view their previous data entries may mean increased potential for unauthorized outsiders to also access this data. Time constraints also influenced the nature and quality of the security system. For example, we chose a third-party web server provider, for it would have taken approximately 8 months to implement the application on the hospital server. Additionally, the web application received approval much later than other components of the study intervention. Thus, we began our study without the web-based application and decided all participants should enroll in it in the second half of the intervention timeline. This approach has two advantages: 1) all participants will receive the same intervention and 2) we will be able to evaluate if adding the web-based application enhances the compliance of participants and study outcomes. Future studies should consider these factors (time and “trade-offs”) when designing web-based applications, being prepared to make sacrifices on application functionality or adapt to changes in timelines during implementation.

## Discussion

Advances in information technology open up a new realm of possibilities for health services and clinical research using web-based applications and mobile devices. Healthcare practitioners and researchers must not neglect their ethical duty to protect patient privacy in their pursuit of developing and implementing the next innovative health technology. This paper discusses, and offers some solutions to, the challenges of protecting patient privacy by outlining the security solutions to a web-based, mobile lifestyle intervention for obese children and adolescents with CHD, and their strengths and weaknesses against threats such as misuse of patient identities and unauthorized access to PI.

In reporting our process, we demonstrate dedication to our ethical duty to protect patient privacy. This transparency and reporting of data security in clinical research can help keep researchers accountable, while also sharing strategies on how to address security and privacy issues. For example, Stopczynski et al. recommends openness amongst researchers collecting data using sensor networks so that new security platforms do not have to be created for every new study [12]. Even in the private sector, Albrecht et al. calls for a standard reporting mechanisms for medical smartphone applications, especially those regarding security, to foster transparency and help users make informed choices [13]. Similarly, we call upon clinical researchers to report on data security when publishing research on web-based and mobile health technology.

Each health intervention is unique as they differ in their purpose, target population, and methods for patient engagement. Thus, the security challenges will be unique. For example, in the Smart Heart Trial, there is no text message component, and consequently, no additional security risks. In contrast, Branson et al. sent text message reminders for appointments to adolescent outpatients, and employed an abbreviation technique (e.g., “C u Wed at 8”) to protect patient confidentiality. As solutions to unique security challenges are shared and reported, perhaps security and privacy developments will adapt in parallel to the proliferation of web-based, mobile technology in healthcare, capturing both ethical merit and innovation in the future of health technology.

Of course, it is still an open controversy as to what extent the PHI is placed at risk using electronic records. The interested reader can read more about the objective measures of risk in (Weston [14]) and more recent reports by the Ontario Director of Health Policy for a Canadian perspective (Grant and Di Re [15]). There will always be some level of risk associated with electronic record keeping, but properly implementing currently available technologies and defence strategies will minimize this risk to an acceptable level.

## Additional file

Additional file 1: Microsoft Word Document. Secure Host address SSL: Description of the Secure Socket Layer (SSL) used for data connections. (DOCX 16 kb)

## Abbreviations

CHD: Congenital heart disease
CIHR: Canadian institutes of health research
IT: Information technology
LHSC: London health sciences centre
MVC: Model, view, controller
NSERC: Natural sciences and engineering research council of Canada
PHI: Personal health information
PHIPA: Personal health information protection act
PI: Personal information
REB: Research ethics board
SSHRC: Social sciences and humanities research council of Canada
TCPS2: Tri-council policy statement
VPN: Virtual private network
