# Supplementary material for: Implementation of clinical research trials using web-based and mobile devices: challenges and solutions
Source: BMC Med Res Methodol. 2017 Mar 17;17:43. doi: 10.1186/s12874-017-0324-6 (PMC5356263; doi:10.1186/s12874-017-0324-6)
Supplement: Additional file 1: — Microsoft Word Document. Secure Host address SSL: Description of the Secure Socket Layer (SSL) used for data connections. (DOCX 16 kb) [file 12874_2017_324_MOESM1_ESM.docx]

***Secure Host address SSL:***

Incoming mail (POP3): pop002.merchantsecure.com

Outgoing mail (SMTP): smtp002.merchantsecure.com

With full access to our Web Application, we have made the following fixes to block direct insecure attacks:

- Within the ASP.NET Application’s root Web.Config file, we have removed the opportunity for attackers to analyze the statistics on error responses, by modifying the <customErrors> section of the web.config file. Note the use of redirectMode=”ResponseRewrite” with .NET 3.5 SP1 and .NET 4.0:

<configuration> <system.web>

<customErrors mode="On" redirectMode="ResponseRewrite" defaultRedirect="~/error.aspx" />

</system.web> </configuration>

- We make use of the following custom C# Error.aspx file, which blocks the ability to use a statistics-based attack by observing different types of error to cracking attempts, by replacing all errors with one single response: “an error occurred while processing your request”.

<%@ Page Language="C#" AutoEventWireup="true" %>

<%@ Import Namespace="System.Security.Cryptography" %>

<%@ Import Namespace="System.Threading" %>

<script runat="server">

   void Page_Load() {

      byte[] delay = new byte[1];

      RandomNumberGenerator prng = new RNGCryptoServiceProvider();

      prng.GetBytes(delay);

      Thread.Sleep((int)delay[0]);

      IDisposable disposable = prng as IDisposable;

      if (disposable != null) { disposable.Dispose(); }

    }

</script>

<html>

<head runat="server">

    <title>Error</title>

</head>

<body>

<div>

        An error occurred while processing your request.

</div>

</body>

</html>
